# Length of Hospital Stay Prediction at the Admission Stage for Cardiology Patients Using Artificial Neural Network

**DOI:** 10.1155/2016/7035463

**Published:** 2016-04-07

**Authors:** Pei-Fang (Jennifer) Tsai, Po-Chia Chen, Yen-You Chen, Hao-Yuan Song, Hsiu-Mei Lin, Fu-Man Lin, Qiou-Pieng Huang

**Affiliations:** ^1^Department of Industrial Engineering and Management, National Taipei University of Technology, Taipei 10608, Taiwan; ^2^Division of Health Insurance, Mackay Memorial Hospital, Taipei 10449, Taiwan; ^3^Medical Affairs Department, Mackay Memorial Hospital, Taipei 10449, Taiwan; ^4^Registration and Admitting, Mackay Memorial Hospital, Taipei 10449, Taiwan

## Abstract

For hospitals' admission management, the ability to predict length of stay (LOS) as early as in the preadmission stage might be helpful to monitor the quality of inpatient care. This study is to develop artificial neural network (ANN) models to predict LOS for inpatients with one of the three primary diagnoses: coronary atherosclerosis (CAS), heart failure (HF), and acute myocardial infarction (AMI) in a cardiovascular unit in a Christian hospital in Taipei, Taiwan. A total of 2,377 cardiology patients discharged between October 1, 2010, and December 31, 2011, were analyzed. Using ANN or linear regression model was able to predict correctly for 88.07% to 89.95% CAS patients at the predischarge stage and for 88.31% to 91.53% at the preadmission stage. For AMI or HF patients, the accuracy ranged from 64.12% to 66.78% at the predischarge stage and 63.69% to 67.47% at the preadmission stage when a tolerance of 2 days was allowed.

## 1. Introduction

The demand for health care services continues to grow as the population in most developed countries ages. To make health care more affordable, policy makers and health organizations try to align financial incentives with the implementation of care processes based on best practices and the achievement of better patient outcomes. The length of stay (LOS) in hospitals is often used as an indicator of efficiency of care and hospital performance. It is generally recognized that a shorter stay indicates less resource consumption per discharge and cost-saving while postdischarge care is shifted to less expensive venues [1]. It motivates the endeavor to develop a diagnosis-related group (DRG) for patient classification based on the type of hospital treatments in relation to the costs incurred by the hospital. This quality assurance scheme was then linked to the prospective payment system (PPS) and adopted by the federal government in the United States for the Medicare program in 1983. This payment system was found to moderate hospital cost inflation due to a significant decline in the average length of stay (ALOS), which refers to the average number of days that patients spend in hospital [2]. Under the assumption that patients sharing common diagnostic and demographic characteristics require similar resource intensity, the aim of DRG is to quantify and standardize hospital resource utilization for patients [3].

Other than diagnostic attributes, most research focuses on two types of factors to explain the variation in LOS: patient characteristics and hospital characteristics. In examining data for the National Health Service (NHS) in the United Kingdom, the variation in LOS for those over age 65 was consistently larger across all regions [4]. It was observed that the variation in LOS between hospitals was larger compared to that between doctors in the same hospital [5]. Hospital policy in treatment management can also determine LOS. It was found that psychiatrists were able to predict LOS with significant accuracy, but only for patients they treated. Moreover, the prediction by a hospital coordinator involved in all patient treatments was significantly more correlated to the true LOS than psychiatrists' predictions [6]. A comparison of data from 24 hospitals in Japan showed that inpatient capacity and the ratio of involuntary admissions correlated positively to longer LOS [7]. A higher level of caregiver interaction among nurses and physicians, such as communication, coordination, and conflict management, was significantly associated with lower LOS [8].

The ability to predict LOS as an initial assessment of patients' risk is critical for better resource planning and allocation [9], especially when the resources are limited, as in ICUs [10, 11]. Yang et al. considered timing for LOS prediction in three clinical stages for burn patients: admission, acute, and posttreatment. Using three different regression models, the best mean absolute error (MAE) in the LOS predictions was around 9 days in both the admission and the acute stage and 6 days in the posttreatment stage. With three more treatment-related variables, the results showed that the prediction accuracy was significantly improved in the posttreatment stage [11]. An accurate prediction of LOS can also facilitate management with higher flexibility in hospital bed use and better assessment in the cost-effectiveness treatment [12, 13].

This prediction can even stratify patients according to their risk for prolonged stays [14, 15]. Spratt et al. used a multivariate logistic regression method to identify factors associated with prolonged stays (>30 days) for patients with acute ischemic stroke. In addition to advanced age (>65), diabetes and in-hospital infection were significantly associated with prolonged LOS [14]. Lee et al. analyzed LOS data on childhood gastroenteritis in Australia and, using either the robust gamma mixed regression or linear mixed regression method, found that both gastrointestinal sugar intolerance and failure to thrive significantly affected prolonged LOS [16]. Schmelzer et al. used the multiple logistic regression method and found that both the American Society of Anesthesiologists (ASA) scores and postoperative complications were significant in the prediction of prolonged LOS after a colectomy [17].

Rosen et al. studied the LOS variation for Medicare patients after coronary artery bypass graft surgery (CABG) in 28 hospitals. They found that including deceased patients did not significantly influence the results. Other than age and gender, the most powerful predictors were history of mitral valve disease or cerebrovascular disease and preoperative placement of an intra-aortic balloon pump. Different hospitals varied significantly in their LOS, and the readmission rate was linearly related to longer LOS [18]. Janssen et al. constructed a logistic regression model to predict the probability for patients requiring 3 or more days in ICU after CABG. Only 60% of the patients predicted to be high risks had a prolonged ICU stay [15]. Chang et al. identified that, among preoperative factors, age of more than 75 years and having chronic obstructive pulmonary disease (COPD) were associated with increased LOS for patients who underwent elective infrarenal aortic surgery [19].

Even though diagnosis had been considered the primary factor affecting hospital stays, patients' clinical conditions, such as the number of diagnoses and the intensity of nursing services required, might be as critical in determining LOS variations within some DRGs [20]. One study showed that only 12% of the variation could be explained by patient characteristics and general hospital characteristics for patients with a primary diagnosis of acute myocardial infarction (AMI) [21]. For heart failure patients, Whellan et al. studied data from 246 hospitals for admission predictors for LOS. Patients with longer LOS had a higher disease severity and more comorbidities, such as hypertension, cardiac dysrhythmias, diabetes mellitus, COPD, and chronic renal insufficiency or failure. However, the overall model based on characteristics at the time of admission explained only a modest amount of LOS variation [22].

The purpose of this study is to develop artificial neural network (ANN) models to predict LOS for inpatients with one of the three primary diagnoses: coronary atherosclerosis (CAS), heart failure (HF), and acute myocardial infarction (AMI) in a cardiovascular unit in a Christian hospital in Taipei, Taiwan. A better recognition in critical factors before admission that determine LOS, or a capacity to predict an individual patient's LOS, could promote the development of efficient admission policy and optimize resource management in hospitals. This study aims to use ANN to predict LOS for patients with three primary diagnoses: coronary atherosclerosis (CAS), heart failure (HF), and acute myocardial infarction (AMI) in a cardiovascular unit. Moreover, two stages in LOS prediction are presented: one uses all clinical factors, designated as the predischarge stage, and the other uses only factors available before admission, designated as the preadmission stage. The prediction results obtained at the predischarge stage are then used to evaluate the relative effectiveness in predicting LOS at the preadmission stage.

The remainder of this paper is organized as follows. In Section 2, the method including steps in data collection and processing and prediction model construction is introduced. Then, the prediction results of various artificial neural network (ANN) models are presented in Section 3. The discussion of the results and the conclusion of the research finding is given in Section 4, with the limitations and future research directions.

## 2. Method

### 2.1. Data Sources and Data Preprocessing

 This study was approved by the Mackay Memorial Hospital Institutional Review Board (IRB) for protection of human subjects in research. Clinical and administrative data were obtained for cardiology patients discharged between October 1, 2010, and December 31, 2011, in a Christian hospital with two locations in the metropolitan area of Taipei, Taiwan: Taipei branch and Tamshui branch. A total of 2,424 admission cases were collected for patients with one of three primary diagnoses: CAS, HF, and AMI. Then 47 admissions were identified as outliers, with more than three standard deviations from the mean, when fitting for both forward addition regression and backward elimination regression models. For the remaining 2,377 cases, 933 were coronary atherosclerosis (CAS) patients, 872 heart failure (HF) patients, and 572 acute myocardial infarction (AMI) patients, as summarized in Table 1. The LOS of any patient in this cardiology unit was defined as from the time of admission to the time of discharge with range from 1 to 35 days, with an average of 5.73, a standard deviation of 5.44, and a median of 4 days. About 63% were male. The age ranges from 21 to 99 years, with an average of 67.07, a standard deviation of 14.35, and a median of 68; 35% were 75 years or older.

An admission case might have zero to multiple comorbidities and similar medical histories were aggregated into comorbidity factors. For example, the history of hypertensive disease includes four types of diseases as identified by ICD-9 codes 401 (essential hypertension) to 404 (hypertensive heart and chronic kidney disease). Each case might have zero to multiple interventions during the admission. Out of a total of 46 types of intervention or diagnostic ancillary services found in the dataset, only the top 6 interventions with more than 5% occurrence in the entire dataset were adopted in this study. The last characteristic, TW-DRG pay, was regarding whether the admission case had been reimbursed by the pay-per-case (i.e., TW-DRG) system implemented by the National Health Insurance Administration (NHIA). The NHIA in Taiwan provides a universal health insurance system and covers approximately 99% of the population [23]. Except for using fee-for-service payment system, the NHIA started introducing the first phase of TW-DRG with 164 groups from 2010. Since cases in the same DRG are reimbursed with the same amount, it is to encourage hospitals to improve their financial performance by better utilizing medical resources [24]. Among the data collected, 25% were reimbursed through TW-DRG payment by the NHIA.

### 2.2. Statistical Analysis

Pearson's correlation coefficients were used to study the relationships between LOS and each inpatient's characteristics. As summarized in Table 2, it was observed that all characteristics were significantly correlated with LOS except for the comorbidity of chronic airway obstruction (ICD 496). As for the risk factors, the top three significant positive correlated variables for longer LOS were patients with heart failure (ICD 428) as main diagnosis, who were older and female. It was consistent with the findings about factors related to prolonged LOS from literature: female, increasing age, and comorbidities such as cerebrovascular disease and diabetes mellitus [18, 19, 22]. The top three significant negative correlated variables for longer LOS were patients with coronary atherosclerosis (ICD 414) as main diagnosis, who went through either percutaneous transluminal coronary angioplasty (PTCA) or percutaneous coronary intervention (PCI).

**Figure 1:**
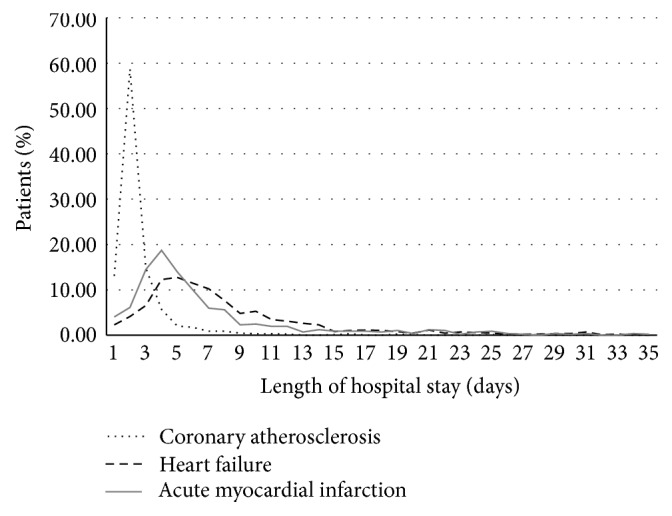
Distribution of LOS for patients for three major diagnoses: CAS, HF, and AMI.

As shown in Figure 1, the distribution in LOS data was skewed with few cases staying longer than 14 days. The average and standard deviation of LOS for CAS patients were 2.63 days and 2.25 days, respectively. For AMI and HF patients, the average and standard deviations of LOS were 7.74 days and 5.93 days, respectively. The distribution of LOS for CAS patients was significantly different than that for patients with either AMI or HF (with *p* value < 0.0001), which suggested different prediction models should be built for CAS patients and for non-CAS patients or referred to as AMI and HF patients.

### 2.3. Structure for Artificial Neural Networks (ANNs)

With the profound growth in clinical knowledge and technology, the development of more sophisticated information systems to support clinical decision making is essential to enhance quality and improve efficiency. Artificial neural networks (ANNs) are useful in modeling complex systems and have been applied in various areas, from accounting to school admission [25]. Walczak and Cerpa proposed four design criteria in artificial neural network (ANN) modeling: the appropriate input variables, the best learning methods, the number of hidden layers, and the quantity of neural nodes per hidden layer [26]. The learning method of ANN can be either supervised or unsupervised, depending on whether the output values should be known before or should be learned directly from the input values. For supervised learning, backpropagation is one of the most commonly used methods due to its robustness and ease of implementation [27].

The clinical benefits of using ANN had been notable in specific areas, such as cervical cytology and early detection of acute myocardial infarction (AMI) [28]. Compared with logistic regression, ANNs were found useful in predicting medical outcomes due to their nature of nonlinear statistical principles and inference [29]. Dybowski et al. adopted an ANN to predict the survival results for patients with systematic inflammatory response syndrome and hemodynamic shock. After improving the performance of ANN iteratively, the predicted outcome was more accurate than using a logistic regression model [30]. Gholipour et al. utilized an ANN model to predict the ICU survival outcome and the LOS for traumatic patients. The results showed that the mean predicted LOS using ANN was not significantly different than the mean of actual LOS [31]. Launay et al. developed ANN models to predict the prolonged LOS (13 days and above) for elder emergency patients (age 80 and over) [32]. Based on the biomedical literature from PUBMED, Dreiseitl and Ohno-Machado showed that the discriminatory performance of ANN models was better or not worse in 93% of the surveyed papers compared to the logistic regression method [33]. Grossi et al. found ANN models outperformed traditional statistic methods in accuracy in various diagnostic and prognostic problems in gastroenterology [34].

The selection of input variables used in an ANN model is critical. Li et al. found that the ANN model using all input variables yielded a slightly higher predictive accuracy than the one using a subset of variables filtered by correlation analysis [35]. Hence, we decided to consider all inpatient characteristics, including gender, age, location, main diagnosis, eight types of comorbidity, six types of intervention, and whether the case met the criteria for TW-DRG reimbursement. These input variables were then categorized into two stages: preadmission stage and predischarge stage, as shown in Table 3. Variables in the preadmission stage included information available prior to hospitalization, such as gender, age, hospital branch (location) to be admitted to, main diagnosis, and comorbidities. In the predischarge stage, additional to variables in the preadmission stage, it includes interventions and whether the case was reimbursed by TW-DRG payment. A case is to be reimbursed by TW-DRG payment, not default pay-per-service, depending on the actual discharge condition such as surgical procedure, treatment, and discharge status according to the NHIA guideline [36].

**Figure 2:**
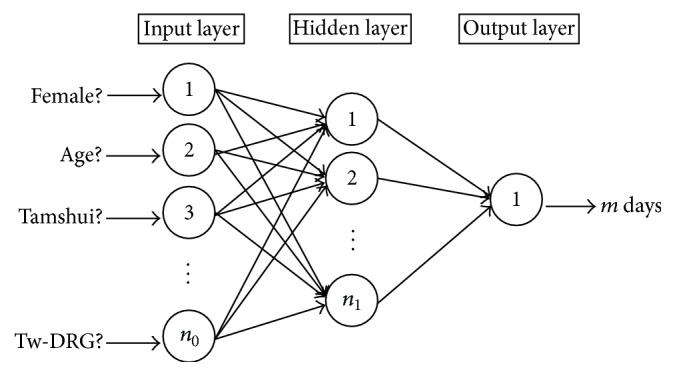
General structure of backpropagation artificial neural networks in this study.

Separate ANNs were built to predict LOS: one for coronary atherosclerosis (CAS) patients and the other for heart failure or acute myocardial infarction AMI and HF patients.  Figure 2 shows the general structure of backpropagation artificial neural networks in this research. The output layer has only one neuron and it generates a number ranged from 0 to 35 to represent the predicted LOS. The size of input layer depends on the number of input variables. Here, the prediction model using input variables in the predischarge stage is referred to as the predischarge model. Likewise, the model using variables in the preadmission stage is referred to as the preadmission model. For a predischarge model, the input layer in an ANN model has 18 neurons (*n*_0_ = 18) for CAS patients. For AMI and HF patients, the value of *n*_0_ is 19 with one additional neuron with Boolean value to represent whether the major diagnosis is HF in a predischarge model. In preadmission models, the value of *n*_0_ is 11 and 12 for CAS patients and for AMI and HF patients, respectively.

As for the hidden layer, more neurons were found to enable a better closeness-of-fit [37] with lower training errors [38]. However, large ANN size also required more training efforts [39] and could result in overfitting [38]. Some research suggested the number of neural nodes in hidden layers to be between 2/3 to 2 times of the size of the input layer [26, 39, 40].

## 3. Results

In this section, the LOS prediction in predischarge model and preadmission model using ANNs is benchmarked with the results using linear regression (LR) models. All prediction models are implemented using IBM® SPSS® v.21 and IBM SPSS Neural Networks 21. Similar to the preliminary trial run, the original data was separated into training dataset and test dataset. The training dataset included 744 admissions for CAS patients and 1,155 admissions for AMI and HF patients, and the test dataset consisted of 189 admissions for CAS patients and 289 admissions for AMI and HF patients. During training any ANN model, 70% of the training dataset were randomly assigned to the training set and the remaining 30% to the validation set. The training stops when the number of training epochs reaches 2,000 or there is no improvement in validation error for 600 epochs consecutively. For LR models, the entire training dataset was used to generate the linear regression functions.

### 3.1. For CAS Patients

The performance of prediction models is evaluated using the same test dataset. Since the LOS predictions obtained by ANN or LR models are continuous numbers, we further define that a prediction of LOS is considered accurate if the difference is within 1 day from the actual LOS for CAS patients. Moreover, the effectiveness of predictability was measured according to the mean absolute error (MAE) and the mean relative error (MRE), defined as follows:

(1) MAE=∑i=1nY~i−Yin,MRE=∑i=1nY~i−Yi/Yin,

where ${\tilde{Y}}_{i}$ and *Y*_*i*_ are the predicted LOS and actual LOS for the *i*th test data, *i* = 1,2,…, *n*, and *n* is the number of testing instances.

**Figure 3:**
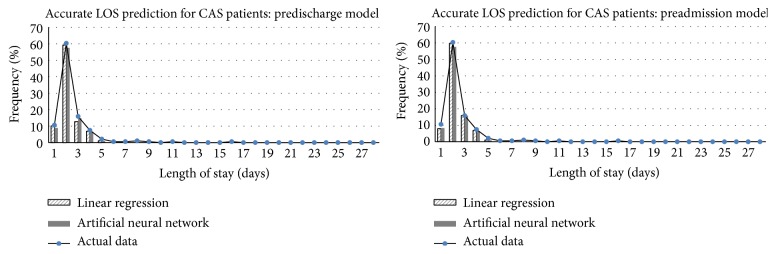
Breakdown of accurate LOS predictions using LR and ANN models for CAS patients in the test data.

To incorporate the randomness in data selected for training ANN, the results showed in Table 4 are the 95% confidence intervals (95% CI) for accuracy, MAE, and MRE based on 30 runs. All models were quite effective in predicting LOS, with the accuracy rate ranging from 88.07% to 91.53%, the MAE from 1 to 1.11 days, and the MRE from 0.44 to 0.47.  Figure 3 shows a detailed look at the distribution in the accurate LOS prediction in the test dataset. It is observed that LR model performed better than ANN model for patients with LOS of 2 days, which was about 60% of the test dataset. However, both LR and ANN models were unable to predict correctly for LOS more than 5 days, which accounted for 3.7% of the test dataset.

### 3.2. For AMI and HF Patients

Same performance indices are used to evaluate the effectiveness of prediction models for AMI and HF patients. Results summarized in Table 5 show that these models are not as effective in predicting LOS as for CAS patients, with the accuracy rate ranging from 32.99% to 36.33%. The MAE of all models has been quite stable, ranging from 3.76 to 3.97 days and the MRE from 0.69 to 0.77. Further, considering the high degree in the variation of LOS distribution, the definition of accuracy has been extended to include two more scenarios: a tolerance of 1 day is allowed (the difference of LOS prediction to the actual LOS is less than 2 days) or a tolerance of 2 days is allowed (the difference of LOS prediction to the actual LOS is less than 3 days). However, the accuracy rate of these models is increased from 63.69% to 67.47% only even with 2 days of deviation in prediction being allowed as in Table 5.

**Figure 4:**
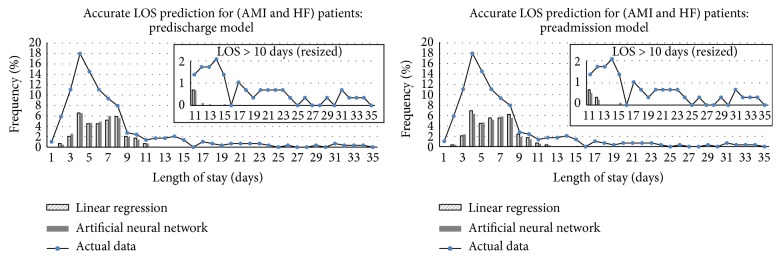
Breakdown of accurate LOS predictions (no tolerance) using LR and ANN models for AMI and HF patients in the test data.

Figure 4 shows the breakdown in the accurate LOS prediction with no tolerance in the test dataset. It is observed that both LR and ANN models performed better in predicting LOS between 8 and 11 days. In the predischarge model, ANN performs better than LR model for patients with LOS of 3, 5, 6, or 7 days, which is about 60% of the test dataset. Moreover, as shown in the resized charts in Figure 4, ANN models were able to predict correctly for cases with LOS greater than 11 days, which accounts for 14.5% of the test dataset. However, both LR and ANN models were unable to predict correctly for LOS greater than 18 days, which accounts for 5.9% of the test dataset.

### 3.3. Validation of ANN Models

To determine a proper structure for ANN used in this study, a preliminary trial run was first conducted to identify a proper structure for ANN models while assuming that the neuron activation function used for each neuron in the hidden layer was log-sigmoidal function with outputs between 0 and 1 [41]. The original data was separated into two sets: training dataset and test dataset. The training dataset included the first 12-month data, from October 1, 2010, to September 30, 2011, with 744 admissions for CAS patients and 1,155 admissions for AMI and HF patients. The test dataset consisted of the data in the last 3 months, with 189 admissions for CAS patients and 289 admissions for AMI and HF patients.

**Figure 5:**
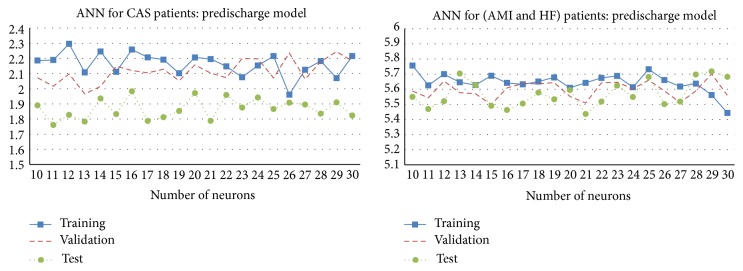
The RMSE in training, validation, and test of trained ANN models with 10 to 30 hidden neurons.

To avoid overfitting, the training dataset was further separated into two sets: a training set, to update the weights and biases, and a validation set, to stop training when the ANN might be overfitting. In this study, the size of training set and validation set in training all ANN models was assumed to be 70% and 30% of the training dataset. The weights in an ANN were modified using a variable learning rate gradient decent algorithm with momentum [42]. The training stopped when the number of training epochs reached 2,000 or there is no improvement in the validation error for consecutively 600 epochs. After an ANN was trained, the model was then used to obtain the predicted LOS in the test dataset. Furthermore, to avoid the effect of randomness when comparing the results, a fixed training set and validation set were used when training the backpropagation ANNs.  Figure 5 shows the root mean squared error (RMSE) for the training set, validation set, and test dataset of trained ANN models with different numbers of neurons in the hidden layer, ranging from 10 to 30. The training errors were found to be slightly decreasing as more neurons were included in the hidden layer. However, no overfitting was observed and the test errors had been quite stable for both models.

**Figure 6:**
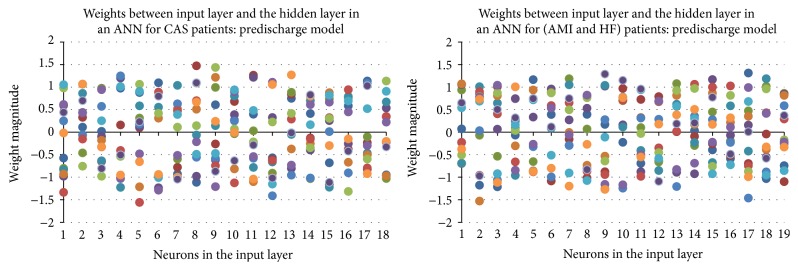
Weight distribution of trained ANN in predischarge model with 13 hidden neurons based on one run.

To balance between the required training effort and the test errors improvement, the number of neurons in the hidden layer, or the value of *n*_1_ in Figure 2, was set to be 13 for all ANN models.  Figure 6 shows the weight distribution between input neurons and hidden neurons, as each dot indicates the weight of one of the input neurons to some hidden neuron, and each input neuron has a total of thirteen dots (weights) linked to the hidden layer. It further validates the size of ANN used in this study since the weights had been scattered evenly from −1.5 to 1.5 with only a few dots (weights) close to zero.

## 4. Discussion and Conclusion

This study proposed the use of the neural network techniques to predict LOS for patients in a cardiovascular unit with one of three primary diagnoses: coronary atherosclerosis (CAS), heart failure (HF), and acute myocardial infarction (AMI). The major observation based on the results was that the preadmission models were as effective in predicting LOS as the predischarge models. It was even found that some preadmission models performed slightly better than predischarge models as shown in Tables 4 and 5. This observation indicates that whether a patient might be reimbursed by TW-DRG did not provide additional predictive ability in LOS, and the assumption that a shorter LOS would be preferred in the sake of hospitals' financial performance when DRG was implemented was not applicable in our case hospital.

The benefit of using ANN models was more significant when predicting prolonged LOS for HF and AMI patients. When predicting prolonged LOS, most literature formulated the prediction models to determine whether an admission might belong to a prolonged stay [14, 15, 17] or whether the LOS might be within a fixed range of LOS days [16, 22]. The study by Mobley et al. [43] predicted the exact LOS days for patients in a postcoronary care unit. With 629 and 127 admissions in the training and test file, a total of 74 input variables were used to predict 1 to 20 LOS days in ANNs. The mean LOS was 3.84 days and 3.49 days in the training file and the test file. They showed no significant difference in the distribution from the predicted LOS and from the actual LOS in the test file. However, ANN with two or three hidden layers made no prediction of LOS beyond 5 days [43]. In this study, the mean LOS was 2.65 days and 2.53 days in the training dataset and the test dataset for CAS patients. With only 18 input variables, our models were able to predict correctly for patients with LOS up to 5 days as shown in Figure 3. For AMI and HF patients, the mean LOS was 7.86 days and 7.23 days in the training dataset and the test dataset. Compared with LR method, the ANN model was able to predict patient stays longer than 11 days, as shown in Figure 4.

In general, it is observed that the LR model performed slightly better than ANN models in terms of accuracy as in Tables 4 and 5. It might be due to the reason that each ANN model was built by only 70% of the training dataset, which consisted of the first 12-month data, and the test dataset, which was the remaining 3-month data, had been highly consistent with the previous 12 months. This phenomenon implies that the clinical pathways were well-established in our case hospital.

Limitation of this research is that the major diagnosis and comorbidities for patients are assumed to be well-known in the preadmission stage. Further study is suggested to fully assess the use of ANN models in LOS prediction, especially for patients who might require longer LOS. Instead of predicting the actual LOS, it might be practical to first categorize LOS into risk groups. More patient characteristics, such as vital signs or lab readings at the time of admission, can be included to improve the performance of LOS predictability.

As the bed supply is limited, the utilization of hospital beds is considered economically critical for most hospitals and any policy related to improving bed utilization has profound impacts on the perception of quality in the provided care and satisfaction of patients and physicians. Currently, hospitalists rely on only aggregated data, such as occupancy rates and average LOS, to access the performance and competitiveness among clinics in the hospital. A reliable LOS prediction in the preadmission stage could further assist in identifying abnormality or potential medical risks to trigger additional attentions for individual cases. It might even allow bed managers to foresee any bottlenecks in bed availability when admitting patients to avoid unnecessary bed transfer between wards.

## Figures and Tables

**Table 1:** Inpatient characteristics, occurrence, and the associated LOS data in this study.

Characteristics		Quantity (*N* = 2377)	Occurrence (%)	LOS (days)		
				Mean	SD	Median
Sex	Male	1501	63	5.05	4.97	5
	Female	876	37	6.89	5.98	7

Age	Less than 65	1059	44	4.42	4.53	3
	65~74	493	21	5.32	5.01	4
	75~84	541	23	7.31	5.83	6
	85 and above	284	12	8.30	6.69	6

Location	Tamshui branch	803	34	6.21	5.39	5
	Taipei branch	1574	66	5.48	5.45	4

Main diagnosis	Coronary atherosclerosis (ICD414)	933	39	2.63	2.25	2
	Heart failure (ICD428)	872	37	8.24	5.87	7
	Acute myocardial infarction (ICD410)	572	24	6.97	5.95	5

Comorbidity	Myocardial infarction (ICD410/412)	134	6	3.99	4.08	2
	Diabetes (ICD250)	954	40	6.24	5.85	4
	Cerebrovascular disease (ICD433/434/437/438)	69	3	7.61	6.14	6
	Cardiac dysrhythmias (ICD427)	447	19	6.81	5.20	6
	Heart failure (ICD428)	394	17	6.27	6.20	4
	Chronic airway obstruction (ICD496)	63	3	5.00	4.21	4
	Hypertensive disease (ICD401/402/403/404)	1218	51	5.07	5.00	3
	Coronary atherosclerosis (ICD414)	909	38	5.39	4.45	4

Intervention	Percutaneous transluminal coronary angioplasty (PTCA)	577	24	4.68	4.71	3
	Percutaneous coronary intervention (PCI)	281	12	4.48	4.61	3
	Coronary angiography	1382	58	3.86	3.78	2
	Coronary stenting	750	32	4.73	4.51	3
	Cardiac catheterization	1297	55	4.01	4.13	2
	Left ventricular X-ray	512	22	3.66	3.98	2

TW-DRG pay	Yes	593	25	2.94	3.09	2
	No	1784	75	6.66	5.73	5

**Table 2:** Pearson's correlation coefficient for each inpatient characteristic to LOS.

Characteristics	Correlation coefficient (*r*)	*p* value
Female sex	0.163^*∗∗*^	0.000

Age	0.251^*∗∗*^	0.000

Location	0.063^*∗∗*^	0.002

Main diagnosis		
Coronary atherosclerosis (ICD414)	−0.459^*∗∗*^	0.000
Heart failure (ICD428)	0.351^*∗∗*^	0.000
Acute myocardial infarction (ICD410)	0.128^*∗∗*^	0.000

Comorbidity		
Myocardial infarction (ICD410/412)	−0.078^*∗∗*^	0.000
Diabetes (ICD250)	0.077^*∗∗*^	0.000
Cerebrovascular disease (ICD433/434/437/438)	0.060^*∗∗*^	0.004
Cardiac dysrhythmias (ICD427)	0.096^*∗∗*^	0.000
Heart failure (ICD428)	0.044^*∗*^	0.032
Chronic airway obstruction (ICD496)	−0.022	0.280
Hypertensive disease (ICD401/402/403/404)	−0.125^*∗∗*^	0.000
Coronary atherosclerosis (ICD414)	−0.050^*∗*^	0.015

Intervention		
PTCA	−0.405^*∗∗*^	0.000
PCI	−0.346^*∗∗*^	0.000
Coronary angiography	−0.125^*∗∗*^	0.000
Coronary stenting	−0.200^*∗∗*^	0.000
Cardiac catheterization	−0.109^*∗∗*^	0.000
Left ventricular X-ray	−0.084^*∗∗*^	0.000

TW-DRG pay	−0.295^*∗∗*^	0.000

(^*∗∗*^ *p* value < 0.01; ^*∗*^ *p* value < 0.05).

**Table 3:** Input variables in preadmission and predischarge stages with associated values for ANN models.

Stage	Variables		Value (Boolean value)	
Preadmission	Gender		Male (0)	Female (1)
	Age		21~99	
	Location		Taipei branch (0)	Tamshui branch (1)
	Main diagnosis (for non-CAS patients)		AMI (0)	HF (1)
	Comorbidity	Myocardial infarction (ICD410/412)	Absence (0)	Presence (1)
		Diabetes (ICD250)	Absence (0)	Presence (1)
		Cerebrovascular disease (ICD433/434/437/438)	Absence (0)	Presence (1)
		Cardiac dysrhythmias (ICD427)	Absence (0)	Presence (1)
		Heart failure (ICD428)	Absence (0)	Presence (1)
		Chronic airway obstruction (ICD496)	Absence (0)	Presence (1)
		Hypertensive disease (ICD401/402/403/404)	Absence (0)	Presence (1)
		Coronary atherosclerosis (ICD414)	Absence (0)	Presence (1)

Predischarge	Intervention	Percutaneous transluminal coronary angioplasty (PTCA)	No (0)	Yes (1)
		Percutaneous coronary intervention (PCI)	No (0)	Yes (1)
		Coronary angiography	No (0)	Yes (1)
		Coronary stenting	No (0)	Yes (1)
		Cardiac catheterization	No (0)	Yes (1)
		Left ventricular X-ray	No (0)	Yes (1)
	Use TW-DRG as payment method		No (0)	Yes (1)

**Table 4:** Results of predischarge and preadmission models for CAS patients.

	Predischarge model		Preadmission model	
	LR	ANN	LR	ANN
Accuracy (%)	89.95%	88.07%~89.64%	91.53%	88.31%~89.65%
MAE	1.09	1.06~1.11	1.00	1.03~1.07
MRE	0.46	0.44~0.47	0.45	0.44~0.47

**Table 5:** Results of predischarge and preadmission models for AMI and HF patients.

		Predischarge model		Preadmission model	
		LR	ANN	LR	ANN
Accuracy (%)	No tolerance	33.91%	34.19%~36.24%	36.33%	32.99%~35.82%
	1-day tolerance	55.36%	50.16%~52.56%	55.71%	49.77%~52.82%
	2-day tolerance	66.78%	64.12%~66.07%	67.47%	63.69%~65.72%

MAE		3.76	3.83~3.91	3.76	3.87~3.97

MRE		0.69	0.71~0.74	0.72	0.73~0.77

## References

[1] OECD (2011). *Average Length of Stay in Hospitals. Health at a Glance 2011*.

[2] Hsiao W. C., Sapolsky H. M., Dunn D. L., Weiner S. L. (1986). Lessons of the New Jersey DRG payment system. *Health Affairs*.

[3] Fetter R. B. (1991). Diagnosis related groups: understanding hospital performance. *Interfaces*.

[4] Martin S., Smith P. (1996). Explaining variations in inpatient length of stay in the National Health Service. *Journal of Health Economics*.

[5] Westert G. P., Nieboer A. P., Groenewegen P. P. (1993). Variation in duration of hospital stay between hospitals and between doctors within hospitals. *Social Science and Medicine*.

[6] Serota R. D., Lundy A., Gottheil E., Weinstein S. P., Sterling R. C. (1995). Prediction of length of stay in an inpatient dual diagnosis unit. *General Hospital Psychiatry*.

[7] Imai H., Hosomi J., Nakao H. (2005). Characteristics of psychiatric hospitals associated with length of stay in Japan. *Health Policy*.

[8] Shortell S. M., Zimmerman J. E., Rousseau D. M. (1994). The performance of intensive care units: does good management make a difference?. *Medical Care*.

[9] Walczak S., Pofahl W. E., Scorpio R. J. (2002). A decision support tool for allocating hospital bed resources and determining required acuity of care. *Decision Support Systems*.

[10] Verduijn M., Peek N., Voorbraak F., de Jonge E., de Mol B. A. J. M. (2007). Modeling length of stay as an optimized two-class prediction problem. *Methods of Information in Medicine*.

[11] Yang C.-S., Wei C.-P., Yuan C.-C., Schoung J.-Y. (2010). Predicting the length of hospital stay of burn patients: comparisons of prediction accuracy among different clinical stages. *Decision Support Systems*.

[12] Lin C.-L., Lin P.-H., Chou L.-W. (2009). Model-based prediction of length of stay for rehabilitating stroke patients. *Journal of the Formosan Medical Association*.

[13] Rowan M., Ryan T., Hegarty F., O'Hare N. (2007). The use of artificial neural networks to stratify the length of stay of cardiac patients based on preoperative and initial postoperative factors. *Artificial Intelligence in Medicine*.

[14] Spratt N., Wang Y., Levi C., Ng K., Evans M., Fisher J. (2003). A prospective study of predictors of prolonged hospital stay and disability after stroke. *Journal of Clinical Neuroscience*.

[15] Janssen D. P. B., Noyez L., Wouters C., Brouwer R. M. H. J. (2004). Preoperative prediction of prolonged stay in the intensive care unit for coronary bypass surgery. *European Journal of Cardio-Thoracic Surgery*.

[16] Lee A. H., Gracey M., Wang K., Yau K. K. W. (2005). A robustified modeling approach to analyze pediatric length of stay. *Annals of Epidemiology*.

[17] Schmelzer T. M., Mostafa G., Lincourt A. E. (2008). Factors affecting length of stay following colonic resection. *Journal of Surgical Research*.

[18] Rosen A. B., Humphries J. O., Muhlbaier L. H., Kiefe C. I., Kresowik T., Peterson E. D. (1999). Effect of clinical factors on length of stay after coronary artery bypass surgery: results of the cooperative cardiovascular project. *American Heart Journal*.

[19] Chang J. K., Calligaro K. D., Lombardi J. P., Dougherty M. J. (2003). Factors that predict prolonged length of stay after aortic surgery. *Journal of Vascular Surgery*.

[20] Berki S. E., Ashcraft M. L. F., Newbrander W. C. (1984). Length-of-stay variations within ICDA-8 diagnosis-related groups. *Medical Care*.

[21] Chen E., Naylor C. D. (1994). Variation in hospital length of stay for acute myocardial infarction in Ontario, Canada. *Medical Care*.

[22] Whellan D. J., Zhao X., Hernandez A. F. (2011). Predictors of hospital length of stay in heart failure: findings from get with the guidelines. *Journal of Cardiac Failure*.

[23] Wen C. P., Tsai S. P., Chung W.-S. I. (2008). A 10-year experience with universal health insurance in Taiwan: measuring changes in health and health disparity. *Annals of Internal Medicine*.

[24] Chen J.-C., Tsai P.-F., Lin F.-M. Simulation study on the effect of diagnosis related group design in length-of-stay and case-mix index for hospitals in Taiwan.

[25] Paliwal M., Kumar U. A. (2009). Neural networks and statistical techniques: a review of applications. *Expert Systems with Applications*.

[26] Walczak S., Cerpa N. (1999). Heuristic principles for the design of artificial neural networks. *Information and Software Technology*.

[27] Medsker L. R., Liebowitz J. (1993). *Design and Development of Expert Systems and Neural Networks*.

[28] Lisboa P. J. G. (2002). A review of evidence of health benefit from artificial neural networks in medical intervention. *Neural Networks*.

[29] Tu J. V. (1996). Advantages and disadvantages of using artificial neural networks versus logistic regression for predicting medical outcomes. *Journal of Clinical Epidemiology*.

[30] Dybowski R., Weller P., Chang R., Gant V. (1996). Prediction of outcome in critically ill patients using artificial neural network synthesised by genetic algorithm. *The Lancet*.

[31] Gholipour C., Rahim F., Fakhree A., Ziapour B. (2015). Using an artificial neural networks (ANNS) model for prediction of intensive care unit (ICU) outcome and length of stay at hospital in traumatic patients. *Journal of Clinical and Diagnostic Research*.

[32] Launay C. P., Rivière H., Kabeshova A., Beauchet O. (2015). Predicting prolonged length of hospital stay in older emergency department users: use of a novel analysis method, the Artificial Neural Network. *European Journal of Internal Medicine*.

[33] Dreiseitl S., Ohno-Machado L. (2002). Logistic regression and artificial neural network classification models: a methodology review. *Journal of Biomedical Informatics*.

[34] Grossi E., Mancini A., Buscema M. (2007). International experience on the use of artificial neural networks in gastroenterology. *Digestive and Liver Disease*.

[35] Li J.-S., Tian Y., Liu Y.-F., Shu T., Liang M.-H. (2013). Applying a BP neural network model to predict the length of hospital stay. *Health Information Science: Second International Conference, HIS 2013, London, UK, March 25–27, 2013. Proceedings*.

[36] Bureau of National Health Insurance (2014). *TW-DRGs Improve Healthcare Quality, Efficiency and Fairness*.

[37] Barnard E., Wessels L. F. A. (1992). Extrapolation and interpolation in neural network classifiers. *IEEE Control Systems*.

[38] Lawrence S., Giles C. L., Tsoi A. (1996). What size neural network gives optimal generalization? Convergence properties of backpropagation.

[39] Boger Z., Guterman H. Knowledge extraction from artificial neural networks models.

[40] Karsoliya S. (2012). Approximating number of hidden layer neurons in multiple hidden layer BPNN architecture. *International Journal of Engineering Trends and Technology*.

[41] Cybenko G. (1989). Approximation by superpositions of a sigmoidal function. *Mathematics of Control, Signals, and Systems*.

[42] Dao V. N. P., Vemuri R. (2002). A performance comparison of different back propagation neural networks methods in computer network intrusion detection. *Differential Equations and Dynamical Systems*.

[43] Mobley B. A., Leasure R., Davidson L. (1995). Artificial neural network predictions of lengths of stay on a post-coronary care unit. *Heart & Lung*.

